# The impact of Pleurotus eryngii on myofibrillar protein: Physicochemical properties and structural alterations in quick-frozen pork patties during freeze-thaw cycles

**DOI:** 10.1016/j.fochx.2025.103064

**Published:** 2025-09-23

**Authors:** Yuanlong Sun, Xiaoze Li, Huanhuan Liu, Yangyang Chai, Yihong Bao, Fangfei Li

**Affiliations:** a College of Life Science, Northeast Forestry University, Harbin, Heilongjiang 150040, China; b Key Laboratory of Forest Food Resource Utilization in Heilongjiang Province, Harbin, Heilongjiang 150040, China

**Keywords:** Pleurotus eryngii, Myofibrillar protein, Physicochemical properties, Structure, Freeze-thaw cycle

## Abstract

This study investigated the impact of Pleurotus eryngii (PE) on the oxidative stability and gel characteristics of myofibrillar protein (MP) from pork patties undergoing multiple freeze-thaw (F-T) cycles. Pork patties with PE served as the experimental group, and those without PE as the control group. The results suggested that PE effectively inhibited MP degradation based on the MP function and structure. After 5 F-T cycles, the protein solubility of MP from patties containing 0.5 % PE powder was significantly higher than the control (*P* < 0.05). Additionally, the inclusion of PE in patties could inhibit the decrease in whiteness and water-holding capacity, the WHC of MP gels in the control group started at 77.05 % and decreased to 48.67 % after 5 F-T cycles. In the experimental group, the WHC started at 83.81 % and decreased to 59.06 % after 5 F-T cycles. Scanning electron microscopy (SEM) images revealed that the freezing and thawing processes disrupted the regular aggregated structure of MP gels. However, MP gel from patties containing PE formed a relatively smoother and denser network after the same F-T cycle. Furthermore, the inclusion of PE inhibited the degradation and structural unfolding of protein. After many F-T cycles, the carbonyl content of MP grew significantly from 1.85 nmol/mg to 11.69 nmol/mg. When 0.5 % of Pleurotus eryngii was added, the carbonyl content increased from 1.72 nmol/mg to 6.67 nmol/mg, the sulfhydryl content in the control and experimental groups decreased from 12.80 nmol/mg and 12.85 nmol/mg to 3.16 nmol/mg and 10.46 nmol/mg, respectively. These findings underscore the significance of PE in maintaining the structural stability of MP and the physicochemical properties of pork patties during F-Tcycles.

## Highlights

• Repeated F-T cycles significantly deteriorated the physicochemical properties of MP.
• The addition of PE effectively mitigated MP gel aggregation induced by F-T cycles.
• Pleurotus eryngii enhanced the quality of MP gels during F-T cycles.
• Pleurotus eryngii inhibited MP oxidation and protected MP structural integrity.

## 1 Introduction

Pork patties products are widely recognized in international markets for their balanced nutrient composition and versatile cooking methods. Freezing is the most prevalent preservation method utilized in the storage and transportation of meat products due to its effectiveness in inhibiting microbial growth and decreasing enzymatic activity (Hertrich & Niemira, 2021). However, ice crystal formation and recrystallization induced by repeated freezing and thawing processes in the process of frozen storage altered the physical and chemical structures of myofibrillar protein (MP). This change leads to the decrease of a* value and sulfhydryl value of meat, softening of texture, increase of carbonyl value (Leygonie et al., 2012) and thiobarbituric acid reactants (TBARS) value (Xu et al., 2021).

MP, which constitutes 55–60 % of total muscle protein, serves as the primary functional component in muscle tissue. These proteins play a central role in shaping the textural, structural, and sensory qualities of muscle-based foods (Chen et al., 2021). Cryostorage-induced protein denaturation may be associated with factors like mechanical damage caused by water migration, protein oxidation (Zhang et al., 2022). It has been shown that F-T cycles cause protein denaturation and damage to muscle fibers (Zhang et al., 2017), and significant changes in secondary and tertiary structures (Feng et al., 2020). Consequently, strategies to mitigate these changes are a significant focus of meat science research.

In recent years, a variety of food additives have been widely applied in the processing of frozen meat products (Ustun & Turhan, 2020). As a kind of natural plant sources, edible mushrooms have attracted growing interest due to their health benefits and functional properties (Kour et al., 2022). In particially, edible mushrooms are rich in protein, dietary fiber, polysaccharides and polyphenols, making them a promising natural antioxidant in food application (Wu et al., 2023). The polysaccharides from Flammulina velutipes and Lentinus edodes can inhibit water distribution and improve texture in marinated meat (Li et al., 2024; Sheng et al., 2024). Lentinula edodes can also improve the taste of sausage (Wang et al., 2019).

Pleurotus eryngii, which is widely cultivated as an important edible mushroom, possess high nutritional and medicinal values. In particular, the natural cryoprotective property of Pleurotus eryngii is of great importance in the preservation of meat (Zhang et al., 2024). However, the mechanism of regulating the quality deterioration of frozen meat products by adding Pleurotus eryngii is still unclear. The motif of our current research was to investigate the impact of Pleurotus eryngii on the MP physicochemical properties obtained from patty during F-T cycles by evaluating the gel quality and protein solubility and turbidity. In addition, the structural integrity and alterations of MP was reflected by the changes in primary, secondary and tertiary structures of MP.

## 2 Materials and methods

### 2.1 Materials and chemicals

Pork shoulder and neck, were sourced from Biyoute Commercial Supermarket in Harbin, Heilongjiang, China. Other chemicals such as hydrochloric acid, trichloroacetic acid, BSA, Ttis-Gly, DTNB were purchased from Macklin (Beijing, China). All chemicals were of analytical grade.

### 2.2 Preparation of Pleurotus eryngii powder

Pleurotus eryngii was dried at 50 °C for 24 h, ground into powder, and then passed through a 100-mesh screen to ensure uniform particle size. The resulting powder was stored in a desiccator until further use.

### 2.3 Sample preparation

Visible connective tissue, adipose tissue and fascia were removed from the pork before it was minced. The minced pork was mixed thoroughly with 12 % ice water and Pleurotus eryngii powder (0 or 0.5 %) (0.5 % is the best addition through preliminary experiment) for 5 min to form patties, each weighing approximately 50 g. Subsequently, the patties were stored at −18 °C. A total of 24 samples were divided into two groups: a control group (*n* = 12) without Pleurotus eryngii powder and an experimental group (n = 12) containing 0.5 % Pleurotus eryngii powder. The samples were frozen at −20 °*C. prior* to analysis, the samples were thawed at 4 °C for 12 h. All measurements were performed in triplicate, and the average value was recorded. The remaining samples were kept frozen at −20 °C for the next cycle. Protein concentration was determined using the biuret method.

### 2.4 MP extraction and MP gel preparation

The minced pork was homogenized 3 times with 4 volumes of cold extraction buffer (10 mM Na_2_HPO_4_· 12 H_2_O, 10 mM NaH_2_PO_4_· 2 H_2_O, 1 mol/L EGTA, 0.1 M NaCl, 2 mol/L MgCl_2_) (pH 7.0) and centrifuged at 3500 ×*g* at 4 °C for 10 min. The pellet obtained was washed 3 times with 4 volumes of extraction buffer (0.1 M NaCl), followed by another centrifugation at 3500 ×*g* at 4 °C for 10 min (Li, Mei, & Xie, 2021). The MP solution was diluted to a concentration of 40 mg/mL using PIPES buffer and transferred to a glass vial. It was then heated in a water bath at 75 °C for 30 min. After heating, the MP gel was cooled to room temperature using ice water. The MP gel was subsequently stored in a refrigerator at 4 °C.

### 2.5 Effects of Pleurotus eryngii on the physicochemical properties of MP

#### 2.5.1 Turbidity

The absorbance measured at 660 nm of a (1 mg/mL) MP solution represents turbidity (Li, et al., 2022).

#### 2.5.2 Solubility

The solubility of MP was determined according to the method described by Zhang et al. (2023). Briefly, 10 mL of MP solution (1 mg/mL) was taken and centrifuged at 10,000 ×*g* for 20 min. The protein content in the supernatant was quantified and divided by the total protein content, then multiplied by 100 to calculate solubility (%). Biuret method was used to measure the absorbance at 540 nm.

#### 2.5.3 Particle size and zeta potential

The MP sample was dissolved in buffer solution and diluted to a concentration of 1 mg/mL. A proper amount of the solution was transferred to the measurement cell. The particle size of MP sample was determined using the laser particle size analyzer (Malvern Instruments Ltd., Malvern, UK), the zeta potential of MP sample was determined using the zeta potential measuring instrument (SCI-02 A, Huanqiu Hengda., CHINA) (Li, Wang, et al., 2022).

#### 2.5.4 Gel whiteness

The whiteness of the gel was measured by a colorimeter (Du et al., 2023). After measuring the values of L* (brightness), a* (redness/green) and b* (yellowness/blue), the whiteness value is calculated by the following formula:

$$\mathrm{Whiteness}=100-\sqrt{\left(100-L{*}^{2}\right)+a{*}^{2}+b{*}^{2}}$$

#### 2.5.5 Gel water-holding capacity (WHC)

The water-holding capacity (WHC) of MP was measured with the method described by Jiang (Jiang et al., 2021). A portion of the gel was taken and centrifuged at 10,000 ×*g* for 15 min. The WHC of the gel was calculated based on the weight of the gel before and after centrifugation using the following formula:

$$\mathrm{WHC}\left(\%\right)=\left(\frac{W_{0}-W_{1}}{W_{0}}\right)\times 100\%$$

W_0_: weight of gel before centrifugation (g), W_1_: weight of gel after centrifugation (g).

#### 2.5.6 Water distribution

Water distribution in MP gels was evaluated with the method described by Lian (Lian et al., 2023). The gel samples were sectioned into cubic specimens with a uniform dimension of 3 cm × 3 cm × 3 cm. The moisture migration and distribution of the sample were determined by LF-NMR analyzer. Using transverse relaxation time (*T*_2_), the parameters are set to 200 kHz, the RF delay is 0.05 ms, the waiting time is 2000 ms, and the echo time is 0.5 ms.

#### 2.5.7 Microstructure

The microstructure of MP gels was obser*v*ed using a scanning electron microscope (JSM-6700F, Jeol Ltd., Japan).

### 2.6 Pleurotus eryngii influence on MP structural

#### 2.6.1 Carbonyl content

Carbonyl content was determined according to the method described by Zhang et al. (2022) with minor modifications. Briefly, 1 mL of MP solution (2 mg/mL) was pipetted and transferred to each centrifuge tube. For the experimental group, 1 mL of 10 mmol/L 2,4-dinitrophenylhydrazine (DNPH) was added; for the control group, 1 mL of 2 mol/L hydrochloric acid (HCl) was added. All tubes were incubated at room temperature for 1 h. Subsequently, 1 mL of 20 % (*w*/*v*) trichloroacetic acid (TCA) solution was added to each tube, followed by centrifugation at 2000 ×*g* for 5 min. The supernatant was carefully discarded, and the resulting pellet was washed three times with 1 mL of ethyl acetate:ethanol (1:1, *v*/v) per wash to remove unreacted DNPH. After the final wash, 3 mL of 6 mol/L guanidine hydrochloride solution was added to the washed pellet, and the mixture was incubated at 37 °C for 15 min. The samples were then centrifuged at 2000 ×*g* for 3 min, and the supernatant was collected. The absorbance was measured at 370 nm.

The formula for calculating the carbonyl content is:

$$\text{Carbonyl content}\left(\mathrm{nmol}/\mathrm{mg}\right)=\frac{A_{1}-A_{0}}{2.1\times 10^{4}\times C_{\mathrm{pro}}}\times 10^{6}$$

A_1_: Absorbance value of the experimental group; A_0_: Absorbance value of the control group; C_pro_: Protein concentration; Molar extinction coefficient: 2.1 × 10^4^ L/(mol·cm).

#### 2.6.2 Sulfhydryl content

The sulfhydryl content was determined according to the method outlined by (Du et al., 2021) with some modifications, and the absorbance was measured at 412 nm.

#### 2.6.3 Fourier-transformed infrared (FTIR) spectroscopy

The samples were scanned using a Fourier transform infrared (FTIR) spectrophotometer (VECTOR 22, Bruker Co., USA) (Nuerjiang et al., 2023). Spectral measurements were acquired over a wavenumber range of 4000 to 500 cm^−1^, with a resolution of 4 cm^−1^ and a data interval of 1 cm^−1^.

#### 2.6.4 Intrinsic fluorescence spectroscopy

Different MP sample were scanned with a fluorescence spectrophotometer (Leica DM I8, German) (Li et al., 2019). The MP solution was diluted to 1 mg/mL, and put into a four-pass cuvette. The protein solution was excited at 283 nm (slit width was 5 nm), scanning speed was 240 nm/min, and scanning range was 300–400 nm.

### 2.7 Statistical analysis

Data were analyzed by means of analysis of variance (ANOVA) using SPSS software. Means ± standard deviations were considered significant when *P* < 0.05. Generate charts using SigmaPlot 15.0.

## 3 Results and discussion

### 3.1 Effects of Pleurotus eryngii on the physicochemical properties of MP

#### 3.1.1 MP turbidity and solubility

Turbidity serves as a critical indicator of MP aggregation (Wang et al., 2009). Turbidity reflects protein coagulation and particle size. In the absence of coagulation, the particle diameter is small, resulting in low turbidity. As protein aggregation increases, the particle diameter grows, leading to higher turbidity. Therefore, higher turbidity is often correlated with increased aggregation. MP turbidity visually reflects protein aggregation, which in turn affects functionality. The increase in MP turbidity induced by oxidation is attributed to the exposure of hydrophobic amino acid residues, which enhances surface hydrophobicity and promotes further protein aggregation, thereby amplifying turbidity (Wang et al., 2020).

**Fig. 1:**
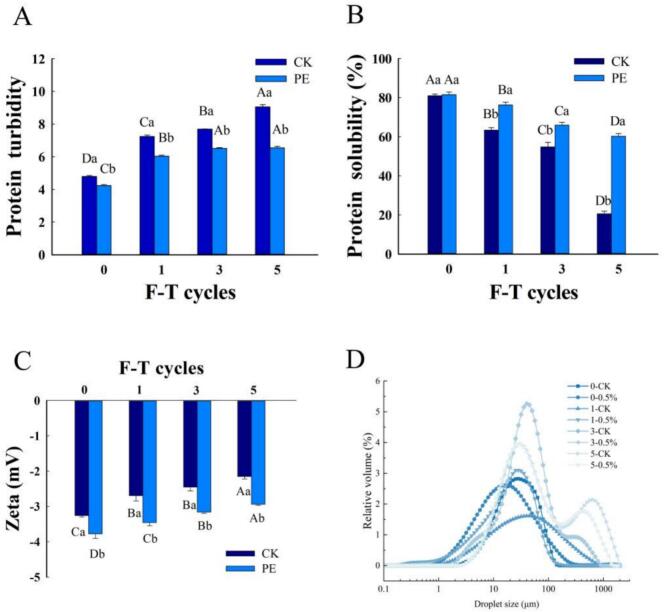
Effects of Pleurotus eryngii powder on turbidity (A), solubility (B), zeta potential (C) and particle size (D) of MP during F-T cycles. PE: Pork patties with PE, CK: pork patties without anything. The means for the same frozen storage with different lowercase letters (a-b) and for the same treatment with different uppercase letters (A-D) differ significantly (*P* < 0.05).

The changes in MP turbidity after different numbers of F-T cycles are shown in Fig. 1A. The addition of Pleurotus eryngii powder to fresh meat significantly lowered the turbidity value of MP in treated groups. Additionally, the turbidity of MP significantly increased in each group with the number of F-T cycles, indicating that the F-T treatment exacerbated the oxidation and aggregation of MP. Moreover, as MP oxidation deepened, it triggered interactions between surface groups, further exposing more hydrophobic groups embedded in the protein molecules. This enhanced the overall hydrophobicity, while the inevitable aggregation of MP resulted in constant collisions, leading to an increase in the absorbance value of the samples and an increase in the turbidity of proteins. Compared to the control group, the turbidity significantly decreased (*P* < 0.05) in the experimental group. At the end of the F-T cycles, turbidity levels were markedly elevated in the control group relative to the Pleurotus eryngii treated samples. This may be attributed to the formation of van der Waals forces, hydrogen bonding, hydrophobic interactions, and electrostatic interactions between the bioactive polysaccharides in Pleurotus eryngii and MP, which collectively enhance the structural stability of MP.

The solubility of MP serves as a key indicator of protein aggregation and denaturation. It is widely regarded as a crucial parameter for characterizing the functional attributes of MP gel (Pan et al., 2024). As depicted in Fig. 1B, with the increase in F-T cycles, a downward trend in MP solubility was observed across all samples. This decline in MP solubility was potentially associated with protein denaturation triggered by the formation and expansion of ice crystals. Such denaturation may stem from protein aggregation due to hydrophobic interactions or the establishment of disulfide and hydrogen bonds, which altered the protein structure during frozen storage and led to MP precipitation. Notably, samples supplemented with Pleurotus eryngii maintained significantly higher MP solubility than control samples across all F-T cycles. Pleurotus eryngii is rich in polysaccharides and phenolic compounds, which can act as cryoprotectants by binding to water molecules, reducing the amount of free water available for ice crystal formation and limiting ice crystal growth during freezing. Ergosterol and phenolic acid in Pleurotus eryngii have also been reported to inhibit protein oxidation by scavenging reactive oxygen species (ROS), thus reducing the oxidative damage to MP structure (Gąsecka et al., 2016) The present study suggested that incorporating Pleurotus eryngii can diminish protein denaturation and aggregation, suppress the growth of ice crystals and mitigate the destruction of cell integrity by larger ice crystals, thereby reducing protein oxidation and denaturation. With the addition of Pleurotus eryngii, ice formation within cells was minimized, and the orderly spatial arrangement between molecules was preserved, which in turn reduced protein aggregation and enhanced protein solubility.

#### 3.1.2 MP zeta potential

The potential of the MP solution is used to analyze the net surface charge of droplets formed by MP. A higher absolute value of the potential indicates greater repulsion between droplets, and a more stable system (Kong et al., 2024). As depicted in Fig. 1C, the zeta potentials for MP were consistently negative, indicating a predominance of negatively charged amino acids in the solution. With the increase in the number of F-T cycles, the negatively charged amino acids surrounding the MP underwent oxidation, leading to a gradual reduction in the absolute value of the zeta potential of MP. This decline signified a progressive decrease in the negative charge on the protein surface. After 5 F-T cycles, the absolute value of the zeta potential reached its minimum, decreasing from the initial −3.77 mV and − 3.26 mV to −2.94 mV and − 2.15 mV respectively. This represented 34.00 % reduction in the control group and a 22.12 % reduction in the treated group. A larger absolute zeta potential contributed to the stability of protein emulsions because the electrostatic repulsion between protein molecules is reduced, thus promoting molecular aggregation. After many F-T cycles, the absolute zeta potential of MP decreased, indicating a deterioration in the electrostatic stability between protein molecules and a tendency for protein aggregation. Nevertheless, the experimental group exhibited a higher absolute value of zeta potential, suggesting improved stability and dispersion of MP. This enhancement can be attributed to the Pleurotus eryngii, which increased molecular repulsion in the protein, thereby improving the stability of the MP structure. Consequently, Pleurotus eryngii improved the stability of MP structure in repeated F-T pork patties (*P* *<* *0.05*).

#### 3.1.3 MP particle size distribution

Particle size serves as an indicator of substance size and aggregation, providing a visual representation of the degree of protein aggregation and affecting the functional properties of proteins (Zhang, Chai, Li, & Bao, 2024). As illustrated in Fig. 1D, MP exhibited a major peak at 0–1 F-T cycle, with the first peak ranging from 10 nm to 100 nm, indicative of small peptides or other protein fragments. At 0 F-T cycles, MP had the largest proportion of small particle sizes. At 3–5 F-T cycles, two major peaks emerged, the second peak ranged from 100 nm to 1000 nm, representing larger aggregates. This indicated that as the number of F-T cycles increased, protein oxidation deepened, the degree of MP aggregation increased.

During the F-T cycles, the average particle size of the samples tended to grow, with the Pleurotus eryngii treatment group having a smaller particle size and a more stable system. At the beginning of the F-T cycles, two groups had relatively uniform particle distributions, indicating stable and homogeneous fresh samples. As the F-T cycles progressed, the average particle size of MP across all samples increased. MP particle size changes were significantly affected by F-T cycles, intensified hydrophobic interactions between proteins facilitated aggregation, thereby enlarging the average particle size. The increase in the number of larger particles clearly indicated the occurrence of protein aggregation. Compared to the control group, the experimental group exhibited a slightly lower quantity of large particles, suggesting that incorporating Pleurotus eryngii into the patties helped mitigate protein aggregation. This effect may be linked with reduced muscle tissue damage. Thus, Pleurotus eryngii can effectively improve the stability of the MP solution system and inhibit the aggregation of MP molecules.

#### 3.1.4 Visualization of MP gel

**Fig. 2:**
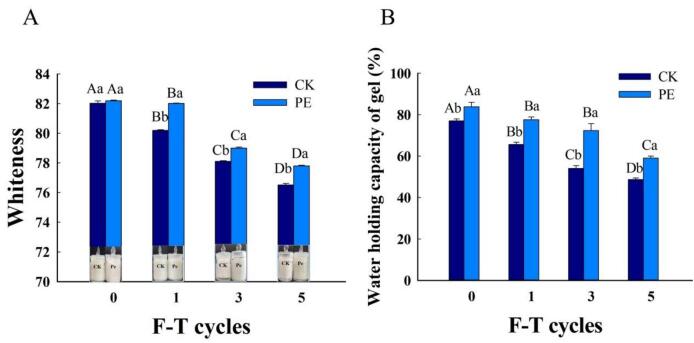
Effect of Pleurotus eryngii powder on whiteness (A), visualization changes (A) and WHC (B) of MP during F-T cycles. PE: Pork patties with PE, CK: pork patties without anything. The means for the same frozen storage with different lowercase letters (a-b) and for the same treatment with different uppercase letters (A-D) differ significantly (*P* < 0.05).

MP formed a gel after heating and cooling, and their apparent morphology can intuitively reflect changes in color, WHC, tissue state and other characteristics of MP during the F-T process (Feng et al., 2023). The appearance of MP gel is illustrated in Fig. 2A. Initially, the MP gel structure was tight, with strong WHC. After many F-T cycles, the gel structure gradually became loose and there was a noticeable decrease in WHC. The overall whiteness of the sample decreased and turned yellow, with water continuously seeping out from the surface. The deterioration of MP was most pronounced at the fifth F-T cycle. Repeated F-T treatments exacerbated the oxidation of collagen MP, leading to decreased WHC and a gradual loosening of the tissue state. Pleurotus eryngii delayed the division of MP gel, improving both color and WHC to varying degrees.

#### 3.1.5 Whiteness and water-holding capacity of MP gel

The effect of the addition of Pleurotus eryngii on the whiteness of MP gel is shown in Fig. 2A. In the control group, the whiteness of the gel significantly decreased (*P* < 0.05) with the extension of storage time, showing a reduction of 6.70 % after 5 F-T cycles. The addition of Pleurotus eryngii inhibited this decrease, resulting in only a 5.30 % reduction in whiteness in the experimental group 5 F-T cycles. The change in whiteness was correlated with the degree of protein denaturation. The decrease in gel whiteness may be attributed to muscle protein oxidation, as well as cross-linking between pigment proteins and myofibrillar proteins induced by lipid oxidation. Additional contributing factors include protein intermolecular interactions and the incomplete removal or residual retention of pigment proteins within the MP. It is also possible that the formation of ice crystals destroys the structure and leads to protein degeneration. This denaturation contributed to a decrease in the quantity of free water within the MP matrix, thus diminishing the gel capacity to reflect light (Yi et al., 2024). Throughout frozen storage, the growth of ice crystals can rupture cells, thereby releasing pro-oxidant substances. These compounds intensified protein denaturation, which in turn contributed to a decrease in the gel whiteness.

WHC is an important indicator of the edible quality of MP gel. After F-T cycles, the WHC of thawed muscle tissue diminished, and the ice crystals generated during frozen storage disrupted the hydration layer of bound water and protein molecules, thereby diminishing the capacity to form stable MP gel (Liu, Tan, Luo, Li, & Hong, 2024). In all samples, WHC decreased, which in turn reduced the gel ability to retain water. As shown in Fig. 2B, the WHC of MP gel in the control group started at 77.05 % and decreased to 48.67 % after 5 F-T cycles. In the experimental group, the WHC started at 83.81 % and decreased to 59.06 % after 5 F-T cycles. Pleurotus eryngii contains polysaccharides that can interact with water molecules, these polysaccharides can act as water holding agents. They have a high affinity for water, which can enhance the water binding capacity within the MP system. Moreover, Pleurotus eryngii is rich in bioactive components such as phenolic compounds. These phenolic compounds can inhibit protein oxidation. Protein oxidation during F-T cycles can lead to changes in protein structure, reducing its ability to bind water. By suppressing oxidation, Pleurotus eryngii helps maintain the integrity of the protein structure, thus preserving the protein's water binding sites and enhancing the overall WHC of the MP gel. These results indicated that incorporating Pleurotus eryngii into the mixture helps reduce water exudation from MP and enhance its WHC.

#### 3.1.6 The water distribution of MP gel

**Fig. 3:**
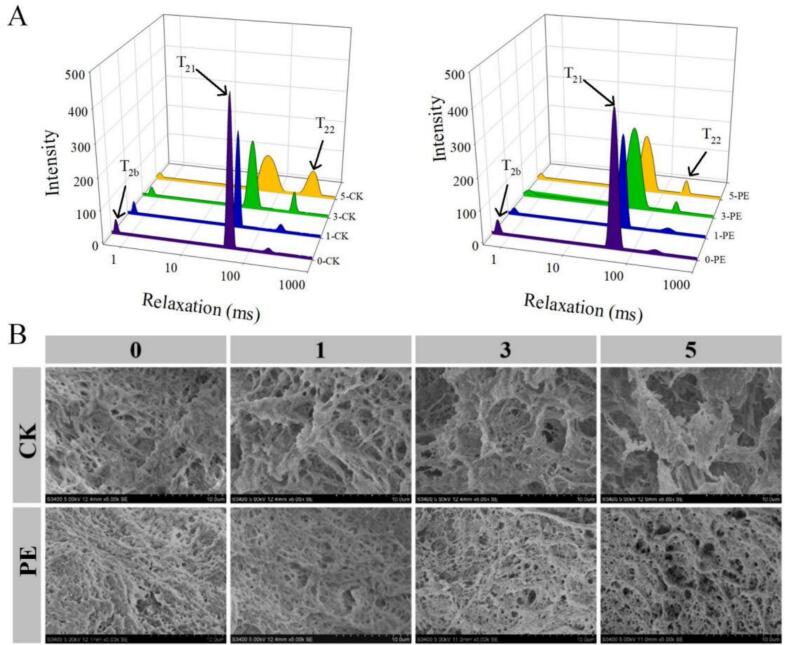
Effect of Pleurotus eryngii powder on the T_2_ relaxation times (A) and on the Scanning electron microscope (C, 5000×) images (B) of MP during F-T cycles. PE: Pork patties with PE, CK: pork patties without anything.

The thermal stability of MP is the key factor to determine the quality and texture of meat products. Low-field nuclear magnetic resonance (LF-NMR) technology is used to analyze the water migration and distribution in the gel network, which can accurately characterize the thermal stability of MP, and then provide theoretical basis for optimizing the processing technology of meat products and improving their sensory characteristics. As depicted in Fig. 3A, the three peaks in the moisture distribution diagram of the gel represented the water trapped in the reticular structure of the MP heat-induced gel (0–10 ms and 10–100 ms), and free water distributed outside the structure of the MP heat-induced gel (100–1000 ms) respectively.

Additionally, the peak area ratios corresponding to different lateral relaxation times (exponential area ratios *P*_21_, *P*_22_, and *P*_23_) provide insights into the relative contents of water in three different states. From Fig. 3A, it can be seen that the addition of Pleurotus eryngii raised the proportion of bound water in MP before the start of F-T treatment. With the increase in the number of F-T cycles, the water distribution state of the gel continuously shifted towards longer relaxation times, indicating a decline in the gel thermal stability and WHC. The gradual conversion of non-fluidizable water in the meat patties to free water corresponds to the decrease in the WHC, primarily due to the hydrolysis of MP and the formation of macromolecular aggregates. The *P*_22_ of the gel gradually decreased and showed a tendency to shift towards *P*_23_. Therefore, after 5 F-T cycles, the immobile water in MP gel was reduced to the minimum (Zhu et al., 2023). Compared to the control group, the Pleurotus eryngii-added group not only reduced the mitigated the prolongation of relaxation time, but also elevated *P*_22_. This strengthened the interaction force between protein and water molecules, thereby increasing the proportion of immobile water and improving the structural properties of MP. Consequently, Pleurotus eryngii effectively delayed the shifting tendency of water distribution in MP gel during repeated F-T cycles of pork patties.

#### 3.1.7 The microstructure of MP gel

The scanning electron micrographs of MP gels are presented in Fig.3B. By examining the microstructure of these gels, we can observe the impact of Pleurotus eryngii on their structural characteristics. At 0 F-T cycles, the addition of Pleurotus eryngii slightly reduced the number of pores in the gel. As the number of F-T cycles increased, the pores in the gel microstructure gradually increased and more irregular and larger pores were gradually produced. After 5 F-T cycles, the three-dimensional mesh structure of the MP gel became increasingly hollow and loose (Liu, Wu, Liang, & Zheng, 2024). Compared to the control samples, the MP gels containing Pleurotus eryngii powder in the experimental group exhibited a more homogeneous and organized structure with smoother surfaces, forming a more continuous and ordered gel structure. The inclusion of Pleurotus eryngii altered the structure of MP, raising the hydrophobic force and water retention capacity, and enhancing protein-protein interactions and viscoelasticity of minced meat. These results suggested that adding 0.5 % Pleurotus eryngii powder was beneficial for maintaining the structural integrity of MP.

### 3.2 MP structural characteristics

#### 3.2.1 The primary structure of MP

The sulfhydryl and carbonyl content of proteins can reflect the integrity of the protein primary structure. The decrease in sulfhydryl content and the increase in carbonyl content of proteins were positively correlated with the increase of protein oxidation, indicating continuous destruction of the primary structure (Wang et al., 2024). Repeated F-T cycles caused irreversible damage to the structure and conformation of MP, impairing protein function, lowering sulfhydryl content and directly oxidizing amino acid side chains, thereby increasing protein carbonyl content. This greatly influences many properties of meat and meat products, MP gradually oxidized, with decreasing sulfhydryl content and increasing carbonyl content. The oxidation of MP was mainly attributed to the release of iron and other pro-oxidant substances as a result of the disruption of cell membranes during F-T treatment. During repeated F-T treatment of pork patties, MP was gradually oxidized with decreasing sulfhydryl content and increasing carbonyl content. The oxidation of MP was mainly attributed to the release of iron and other pro-oxidant substances due to the disruption of cell membranes during the F-T treatment.

**Fig. 4:**
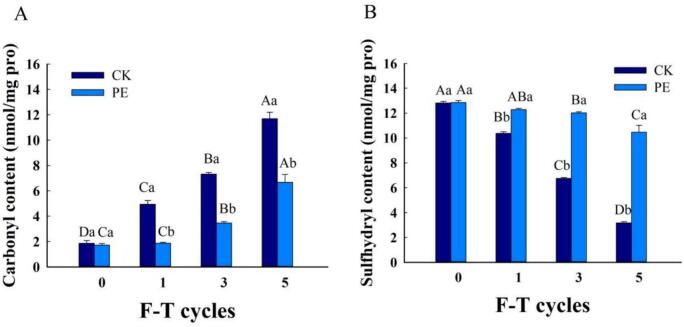
Effects of Pleurotus eryngii powder on Carbonyl Content (A) and Sulfhydryl Content (B) of MP during F-T cycles. PE: Pork patties with PE, CK: pork patties without anything. The means for the same frozen storage with different lowercase letters (a-b) and for the same treatment with different uppercase letters (A-D) differ significantly (*P* < 0.05).

The rise in carbonyl content was driven mainly by the progressive formation of ice during freezing, the increase in the concentration of pro-oxidants, and the subsequent intensification of protein oxidation. Pleurotus eryngii is rich in phenolic compounds with strong free radical-scavenging activity. These phenolics directly quench hydroxyl radicals and superoxide anions generated during F-T cycles, which are key initiators of amino acid side-chain oxidation into carbonyl groups. By virtue of its ability to neutralize these reactive oxygen species (ROS). Pleurotus eryngii effectively interrupts the propagation of protein oxidation chain reactions, thereby exerting a regulatory effect on the excessive accumulation of protein carbonyls. The change in the carbonyl content of MP is illustrated in Fig. 4A. After many F-T cycles, the carbonyl content of MP grew significantly from 1.85 nmol/mg to 11.69 nmol/mg. When 0.5 % of Pleurotus eryngii was added, the carbonyl content increased from 1.72 nmol/mg to 6.67 nmol/mg. This increase in carbonyl content was significantly suppressed by the addition of 0.5 % Pleurotus eryngii, which effectively inhibited MP oxidation, thus effectively disrupting the propagation of the protein oxidation chain reaction, scavenging hydroxyl radicals, and preventing excessive accumulation of carbonyl groups (Wan et al., 2023).

Sulfhydryl groups play a key role in stabilizing the spatial structure of proteins and maintaining their physicochemical and functional properties. MP contain a large number of sulfhydryl groups, which are oxidized into disulfide bonds during the denaturation of MP. For this reason, sulfhydryl content can serve as an indicator of changes in the conformational properties of MP (Li, et al., 2021). During F-T cycling, the spatial structure of proteins was damaged by the formation of ice crystals in muscle tissues, which exposed the buried sulfhydryl groups (-SH) on the protein surface. The continuous oxidation of cysteine sulfhydryl groups on the protein surface resulted in the formation of disulfide bonds, while the oxidation of MP resulted in the production of large reactive carbonyl molecules. Therefore, the carbonyl groups gradually increased, further altering the original spatial structure of proteins. The change of sulfhydryl content in the two groups is shown in Fig. 4B. After 5 cycles, the sulfhydryl content in the control and experimental groups decreased from 12.80 nmol/mg and 12.85 nmol/mg to 3.16 nmol/mg and 10.46 nmol/mg, respectively. This decrease may be attributed to the oxidation of reactive sulfhydryl groups during F-T cycles and the damage of protein structure as a result of ice crystal growth. Some reactive sulfhydryl groups within the protein molecule were oxidized to form disulfide bonds, leading to a reduction in total sulfhydryl groups. As the number of F-T cycles increased, the total sulfhydryl content generally showed a downward trend. The polysaccharides of Pleurotus eryngii interact with MP through hydrogen bonding and hydrophobic interactions, which help stabilize the protein's tertiary conformation and reduce the exposure of internal sulfhydryl groups. Additionally, the phenolic compounds in Pleurotus eryngii scavenge ROS, inhibiting the oxidation of exposed sulfhydryl groups. This finding is consistent with previous studies. The total sulfhydryl content in the control group exhibited a more significant decline compared to the experimental group, suggesting that Pleurotus eryngii better maintained the spatial structure of the protein when complexed with MP.

#### 3.2.2 The secondary structure of MP

**Fig. 5:**
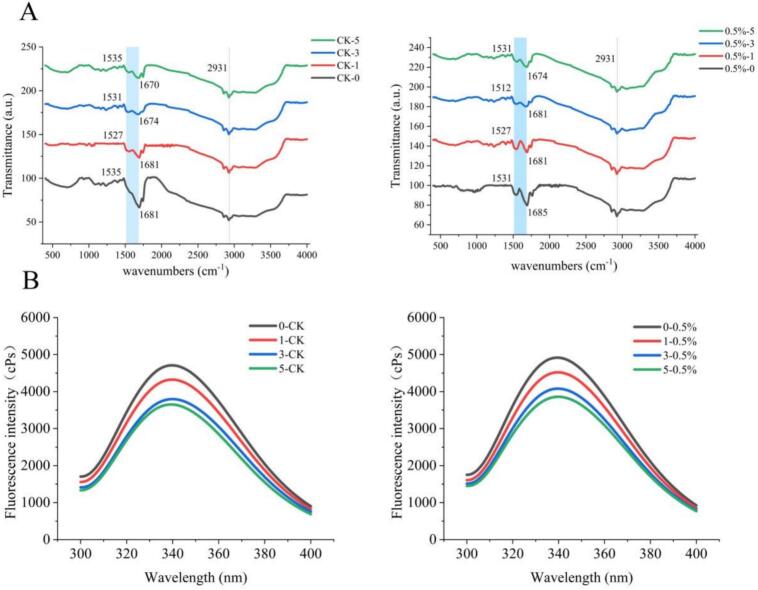
Effect of Pleurotus eryngii powder on the secondary structure (A) and tertiary structure (B) of MP during F-T cycles.

Following infrared spectroscopy, the constituent amide bonds of the excited polypeptide chain absorb energy and undergo vibrations. FTIR can reflect and measure changes in the secondary structure of MP. This is achieved through the amide I region, which ranges from 1600 cm^−1^-1700 cm^−1^. The amide I region's alterations arise from contributions of both C=O and C—N stretching vibrations (Fevzioglu et al., 2020). The FTIR spectra of MP subjected to different numbers of F-T cycles are depicted in Fig.5A. F-T treatment consistently caused the amide I band (associated with the β-folded structure) of the protein to shift towards lower frequencies, and undergo a redshift. Besides, the addition of edible fungi to the protein during five F-T cycles had a positive impact on the functional groups within the MP. In the amide I band, the peak in the control group shifted from 1681 cm^−1^ to 1670 cm^−1^ after five F-T cycles, while the peak in the experimental group fluctuated from 1685 cm^−1^ to 1674 cm^−1^. This implied that the addition of Pleurotus eryngii shifted the amide I band of the protein to a higher frequency. The experimental group demonstrated stronger absorption peaks and a blue shift, particularly in the -C=O- region of the amide, which generated absorption peaks in the spectrum. This shift may be attributed to the breaking and reconstruction of hydrogen bonds, which enhanced the induced effect on MP, increasing the force constants of the chemical bonds and further altering the structure of the functional groups. This resulted in a shift in the absorption frequency towards higher wave numbers. Accordingly, the chemical bonds in the control group weakened, and the atomic mass grew. The secondary structure of protein becomes more unstable with the breaking of hydrogen bonds. The inclusion of Pleurotus eryngii effectively mitigated the deterioration of the structural properties of MP during the mobilization process.

#### 3.2.3 The tertiary structure of MP

Fluorescence spectroscopy provides insights into the tertiary structure of a protein by analyzing the degree of tryptophan quenching in a solution of a given protein concentration. When MP are folded, tryptophan is present mainly in the hydrophobic structure inside the protein, resulting in high fluorescence intensity. However, when MP are partially or completely unfolded, tryptophan is exposed on the surface of protein molecules, resulting in deeper quenching and lower fluorescence intensity (Huang et al., 2022). During the F-T cycles, the fluorescence intensity across all samples gradually decreased with the extension of storage time, suggesting that the protein structure was compromised to a certain extent during freezing and storage. Fig. 5B illustrates the changes in the tertiary structure of MP as a function of the number of F-T cycles in both the control and experimental groups. Compared to unoxidized MP, the fluorescence intensity of MP in samples subjected to repeated F-T cycles was reduced and red-shifted, and the maximum fluorescence emission value of the control group shifted from 339.00 nm to 340.00 nm as the number of F-T cycles increased from 0 to 5. This shift was associated with the oxidation-induced unfolding of the protein tertiary structure, where the binding of tryptophan and tyrosine residues partially shielded fluorescence emission, while the hydrophobicity of the protein grew. Compared to the control group, the addition of Pleurotus eryngii led to an increase in MP fluorescence intensity and a blue shift (the maximum fluorescence emission value for the Pleurotus eryngii group was 339.60 nm after 5 F-T cycles). The fluorescence peak in the experimental group was consistently higher than that of the control group after each F-T cycle, suggesting that Pleurotus eryngii had an inhibitory effect on the oxidation of MP and minimized the exposure of hydrophobic groups. This indicated that the microenvironment of the Pleurotus eryngii-treated group became more hydrophobic, with less exposure of tryptophan in MP, which diminished the decline in the fluorescence intensity of endogenous tryptophan, and its tertiary structure was more complete. On the one hand, polyphenols and polysaccharides in edible mushrooms significantly reduced oxidative degradation of MP during repeated freeze-thaw cycles, thus reducing the exposure of hydrophobic aliphatic groups. On the other hand, the hydrophilic hydroxyl groups in Pleurotus eryngii gradually revealed their effects on MP during the F-T process. A study by Xiankang Fan found that lentinan augmented the α-helix content and the intrinsic fluorescence intensity of tryptophan, while also diminishing the exposure of hydrophobic groups and decreasing protein aggregation (Fan et al., 2023), which aligned with the findings of this study.

## 4 Conclusions

With the increase of the number of F-T cycles, the turbidity, particle size and carbonyl content of MP significantly grew, while solubility, whiteness, WHC and total sulfhydryl content significantly fell. Repeated F-T cycles reduced the intrinsic fluorescence intensity of the samples due to the exposure of tryptophan residues. The experimental group, supplemented with Pleurotus eryngii, alleviated the increase in turbidity, particle size and carbonyl content and inhibited the decrease in solubility, whiteness, water retention and total sulfhydryl content of MP. This suggested that the inclusion of Pleurotus eryngii in appropriate amounts can change the textural properties of MP. These findings offer new insights into the preservation of frozen meat, providing valuable information for future research on the freezing protective effects of Pleurotus eryngii on meat and meat products. Future studies could explore the specific combination principle between PE and MP or PE and ice crystals at the molecular level.

## CRediT authorship contribution statement

**Yuanlong Sun:** Writing – original draft, Validation, Software, Methodology, Investigation, Funding acquisition, Formal analysis, Conceptualization. **Xiaoze Li:** Investigation, Formal analysis. **Huanhuan Liu:** Software, Investigation. **Yangyang Chai:** Resources, Conceptualization. **Yihong Bao:** Resources, Conceptualization. **Fangfei Li:** Writing – original draft, Supervision, Funding acquisition, Conceptualization.

## Declaration of competing interest

The authors declare that they have no known competing financial interests or personal relationships that could have appeared to influence the work reported in this paper.

## Data Availability

Data will be made available on request.
